# Mapping socioeconomic inequalities in malaria in Sub-Sahara African countries

**DOI:** 10.1038/s41598-021-94601-x

**Published:** 2021-07-23

**Authors:** Gabriel Carrasco-Escobar, Kimberly Fornace, Tarik Benmarhnia

**Affiliations:** 1 Health Innovation Laboratory, Institute of Tropical Medicine “Alexander Von Humboldt”, Universidad Peruana Cayetano Heredia, Lima, Peru; 2 Scripps Institution of Oceanography, University of California, San Diego, CA USA; 3 Faculty of Infectious and Tropical Diseases, London School of Hygiene and Tropical Medicine, London, UK; 4 Department of Family Medicine and Public Health, University of California, San Diego, CA USA

**Keywords:** Malaria, Epidemiology

## Abstract

Despite reductions in malaria incidence and mortality across Sub-Saharan (SSA) countries, malaria control and elimination efforts are currently facing multiple global challenges such as climate and land use change, invasive vectors, and disruptions in healthcare delivery. Although relationships between malaria risks and socioeconomic factors have been widely demonstrated, the strengths and variability of these associations have not been quantified across SSA. In this study, we used data from population-based malaria indicator surveys in SSA countries to assess spatial trends in relative and absolute socioeconomic inequalities, analyzed as social (mothers’ highest educational level—MHEL) and economic (wealth index—WI) inequalities in malaria prevalence. To capture spatial variations in socioeconomic (represented by both WI and MHEL) inequalities in malaria, we calculated both the Slope Index of Inequality (SII) and Relative Index of Inequality (RII) in each administrative region. We also conducted cluster analyses based on Local Indicator of Spatial Association (LISA) to consider the spatial auto-correlation in SII and RII across regions and countries. A total of 47,404 participants in 1874 Primary Sampling Units (PSU) were analyzed across the 13 SSA countries. Our multi-country assessment provides estimations of strong socioeconomic inequalities between and within SSA countries. Such within- and between- countries inequalities varied greatly according to the socioeconomic metric and the scale used. Countries located in Eastern Africa showed a higher median Slope Index of Inequality (SII) and Relative Index of Inequality (RII) in malaria prevalence relative to WI in comparison to countries in other locations across SSA. Pockets of high SII in malaria prevalence in relation to WI and MHEL were observed in the East part of Africa. This study was able to map this wide range of malaria inequality metrics at a very local scale and highlighted the spatial clustering patterns of pockets of high and low malaria inequality values.

## Introduction

In the last years, Sub-Saharan African (SSA) countries were on track for reductions in malaria incidence and mortality^1,2^. However, most of worldwide malaria cases (93%) and deaths (94%) still occur in this region, mainly (99.7%) caused by *Plasmodium falciparum*^3^. Previous studies highlighted the strong spatial heterogeneity in malaria transmission between- and within- SSA countries and highlighted the importance of pockets of malaria transmission at the macro- and micro- epidemiological level across stable and unstable malaria foci^4,5^. Ecological composition that favor micro-habitats for breeding sites of dominant malaria vectors (*Anopheles gambiae, Anopheles coluzzii, Anopheles funestus,* and *Anopheles arabiensis*)^6^ is a major driver to the scattered or clustered pattern of malaria transmission^7–10^. However, other important factors collide with such environmental determinants to intensify malaria heterogeneities, such as dwelling infrastructure^11^, human mobility^12,13^, and socioeconomic status^14^.

The malaria elimination efforts are currently facing multiple regional challenges. Climate and land use changes are major Regional health threats that in turn poses critical risks to achieving malaria elimination^15,16^. Changes in meteorological and ecological patterns influence the distribution of the vector, human hosts and alter the biological cycle of the parasite and vector^17,18^, that ultimately, may diverge current epidemiological trends in malaria-endemic countries^19–21^. Such as in other infectious diseases, socioeconomic inequalities were reported in malaria cases and deaths^22–25^. Socioeconomic status may influence malaria risks through multiple different mechanisms. For example, improved housing associated with increased wealth may substantially decrease child malaria risks^26^ and socioeconomic development may be one of the most effective interventions for malaria control^27^. However, the role of socioeconomic factors in reducing malaria risks may vary substantially between and within countries. While agricultural development is frequently associated with increased prosperity, the expansion of irrigated lands may increase vector densities and lead to counterintuitive associations between socioeconomic status and malaria risks^28^. Important evidence about the socioeconomic inequalities on the malaria risk was reported in previous studies done in SSA countries such as in Uganda^22^, Kenya^23,29^, Tanzania^30^, and Nigeria^24^. These studies highlighted the substantial heterogeneity between socioeconomic metrics at a subnational and local level and the utility of their identification to target malaria control interventions. However, no study comprehensively assessed malaria socioeconomic inequalities across many SSA countries at a fine spatial scale.

The Demographic and Health Surveys (DHS) and in particular the Malaria Indicator Survey (MIS) provides information about malaria screening and sociodemographic variables in multiple countries, moreover, when multi-country assessments in the SSA region are scant. This dataset represents a unique opportunity to capitalize on to identify such malaria heterogeneities across the socioeconomic gradient. In particular, the socioeconomic status (SES) implies multiple, yet related, mechanisms that may impact malaria inequalities. Income or wealth reflect the resources to afford the direct and indirect costs of a malaria infection and access to preventive measures for example^31,32^. On the other hand, maternal education (as a social dimension of SES) captures the receptiveness and knowledge capital of the family about preventive measures and early health-seeking behaviors^33,34^. Previous studies highlighted the role of the maternal education on the child’s health in developing countries^35–37^; however, few studies have examined independently the wealth and socioeconomic status inequalities in malaria^38,39^.

Commonly, the relative position in the distribution of wealth or socioeconomic indicator is not comparable across populations (i.e. geographical locations). The slope index of inequality (SII) and the relative index of inequality (RII) are two major indicators for the cross-population comparison and quantification of the wealth/socioeconomic gradient in the relative and absolute scale, respectively. Both indicators provide key and complementary information based on the relative socioeconomic position of the individual in the population, termed `*socioeconomic rank`*^40–43^. Particularly, studies in other infectious diseases such as in HIV showed contrasting results in the inequality interpretation based on the absolute or relative scale of the inequality metric^44,45^. Also, since both indicators compare hypothetical extremes of the socioeconomic rank and are estimated using a regression framework, the RII and SII are considered a summary measure rather than a true population parameter and could be used for comparisons between and within countries^40^. In this study, we used data from population-based malaria indicator surveys in thirteen SSA countries to assess spatial trends in relative and absolute social (mothers’ highest educational level—MHEL) and economic (wealth index—WI) inequalities in malaria prevalence. We aimed to extend these estimations at fine spatial scale in the SSA region. We also aimed to identify clusters of high or low SES malaria inequalities across SSA countries.

## Methods

### Study design and data description

Our study is a multi-country cross-sectional data analysis of the Malaria Indicator Survey (MIS) from thirteen SSA countries for which such data has been collected (Angola, Burkina Faso, Burundi, Kenya, Liberia, Madagascar, Malawi, Mali, Mozambique, Sierra Leone, Tanzania, Togo, and Uganda). We used data from the most recent MIS (from 2015 to 2018), developed by the Monitoring and Evaluation Working Group (MERG) of the Roll Back Malaria initiative. Data of malaria test results, WI, and MHEL were accessed through the DHS Program. The GPS coordinates of the Primary Sampling Units (PSU) were available with an injected 0–2 km displacements in urban clusters, and 0–5 km for rural clusters. In addition, the administrative boundaries were accessed via DHS spatial data repository (https://spatialdata.dhsprogram.com/). Admirative boundaries represent sub-national regions, commonly administrative level 1, and varies between survey years and countries. Two variables were analyzed for relative (i.e. RII) and absolute (i.e. SII) inequalities in malaria prevalence, the WI and MHEL. Inequality estimates were computed and mapped at the PSU and administrative level. For the main analyses, we considered PSU with a sample size higher than 10 and we conducted sensitivity analyses (see details below). In this paper, the main metric of interest is related to SES inequalities in malaria positive tests focusing on two distinct SES measures (WI and MHEL) and two scales to capture such inequalities.

### Exposure and outcome definitions

We defined the malaria test result based on the malaria screening in blood samples conducted by rapid diagnostic test (RDT) during field work. No malaria species identification result was included in this study. A positive malaria test result was considered if the sample was positive to any malaria species, and negative otherwise.

The WI is a composite measure based on a Principal Component Analysis (PCA) of a household's cumulative living standard using household's ownership of selected assets, such as televisions and bicycles; materials used for housing construction; and types of water access and sanitation facilities as described elsewhere^46^. Finally, MHEL was also explored and was re-classified in four categories: no education, elementary/primary, secondary, and higher education. While MHEL was also measured in years of formal education completed by each woman, we reclassified MHEL into these four categories to have subgroups with homogenous sample sizes and reduce the risk of empty cells (i.e. MHEL subgroups with no positive malaria test).

### Socio-economic inequality analysis

Two metrics—on the relative and absolute scales—were used to assess the SES inequalities in malaria prevalence at both the PSU and administrative levels relative to WI and MHEL. The SII and the RII^41^ were computed separately for WI and MHEL. In SII and RII calculation, SES variable was ranked from high to low, thus high values of SII and RII represents high malaria concentration among low-SES levels, and low values represents high malaria concentration among high-SES levels. SII represents the linear regression coefficient that shows the relation between malaria prevalence in each SES level and the hierarchical ranking of SES level. RII expresses the ratio of the predicted outcomes between population in the highest SES level and the lowest SES level. Both indicators were obtained by fitting a linear regression to estimate the coefficient between participants’ relative SES rank and malaria prevalence^41^. In addition, a third inequality metric, the concentration index (CI), was evaluated. CI computation details were presented in the supplementary information. For additional data exploration, Spearman's rank correlation coefficient (Spearman's ρ) was conducted to examine the association between different scales (SII/RII) of the same SES variable, and different SES measures (WI/MHEL) using the same scale of the inequality index.

### Sensitivity analysis

Since the sample size in each PSU varies across countries, extreme malaria prevalence estimates may result from very small sample sizes (Supplementary Fig. 1). Sensitivity analyses were conducted by including only PSU with a sample size higher than 20 and 30.

### Spatial analysis

Spatial autocorrelation of CI, SII, and RII was assessed using local Getis-Ord Gi* statistic (a type of Local Indicator of Spatial Association—LISA) to identify local patterns and clusters of high- and low-inequality across SSA countries. A distance-based neighborhood structure was used for Getis-Ord Gi* computation*.* Neighboring PSUs were defined based on the distance *d* that assigns at least one neighbor to each PSU (nearest neighbor). Gi* statistic was categorized based on the sign (cold- or hotspot) and percentile (90%, 95%, 99%) to prevent bias due to multiple and dependent tests^47^. Data assemble, statistical analyses, spatial data processing, and visualizations were performed in R software v.4.0.1 (R: A language and environment for statistical computing, R Core Team, R Foundation for Statistical Computing, Vienna, Australia (2021) http://www.R-project.org/).

## Results

A total of 47,404 participants (a subset of 33,187 for the analysis of MHEL) in 1,874 (MHEL subset: 1,603) PSUs were analyzed across the 13 SSA countries. The sample size in the PSUs ranges from 10 to 114 (MHEL subset: 10 to 107) participants. Overall, a weighted prevalence of 36.44% was observed in all the SSA countries, ranging from 16.02% in Madagascar to 55.4% in Sierra Leone (Table 1). The sample size and prevalence distribution by PSU is presented in Supplementary Fig. 1.

**Table 1:** Summary statistics of 13 Sub-Saharan African countries.

Country	Sample size	Prevalence*	Wealth ^		Maternal Education ^	
			SII	RII	SII	RII
AO	1,199	22.22%	− 0.014	0.944	0.003	1.013
BF	4,954	20.00%	0.054	1.458	0.156	3.827
BU	2,099	16.67%	0.053	1.455	0.154	3.161
KE	4,318	16.16%	0.083	1.601	0.157	3.161
LB	2,685	52.98%	0.090	1.225	0.197	1.609
MD	1,947	8.08%	0.063	3.110	0.027	1.629
ML	6,534	31.83%	0.040	1.126	0.092	2.029
MW	1,840	33.33%	0.200	2.212	0.222	2.765
MZ	3,430	43.74%	0.131	1.504	0.109	1.400
SL	7,406	60.00%	0.064	1.112	0.084	1.172
TG	2,831	46.97%	0.053	1.123	0.092	1.336
TZ	2,942	13.33%	0.129	2.182	0.114	1.917
UG	5,219	26.06%	0.072	1.391	0.086	1.632

Sample size, prevalence and median slope index of inequality (SII) and relative index of inequality (RII) by country.

*Weighted prevalence; ^ median values.

### SES inequalities in malaria prevalence

Important heterogeneities on SES inequalities on malaria prevalence was observed across the SSA countries. Malawi, Tanzania, and Mozambique were the countries with the greatest median WI inequality in the absolute and relative scales, respectively (Supplementary Fig. 2 and Supplementary Fig. 3). Malawi was the country with the greatest median MHEL inequality in the absolute scale (Supplementary Fig. 4) followed by Liberia in the relative scales (Supplementary Fig. 5). Greater variability in SII for WI was observed in Togo (sd: 0.406) followed by Malawi (sd: 0.397) (Supplementary Fig. 2) and Liberia (sd: 0.397), and for MHEL in Sierra Leone (sd: 0.58) followed by Angola (sd: 0.56) and Malawi (sd: 0.54) (Supplementary Fig. 4). In comparison, the country where the greatest variably in RII for WI was observed is Tanzania (sd: 186.9) followed by Mali (sd: 156.3) and Madagascar (sd: 84.28) (Supplementary Fig. 3), and for MHEL was Burkina Faso (sd: 26.19) followed by Burundi (sd: 22.25) and Liberia (sd: 21.39) (Supplementary Fig. 5).

Overall, SES-inequality showed moderate correlation between WI and MHEL in the absolute (rho-SII: 0.12; p-value < 0.001) and relative (rho-RII: 0.16; p-value < 0.001) scales. Kenya is the only country with a negative correlation between WI/MHEL-SII and the strongest WI/MHEL-SII correlation was observed in Liberia. On the other hand, the strongest correlation in WI/MHEL-RII was observed in Angola, and Kenya, Burkina Faso, and Burundi showed a negative WI/MHEL-RII correlation (Supplementary Table 1). No significant patterns of SES-inequality in both scales (SII or RII) as a function of the PSU prevalence was observed for WI and MHEL (Supplementary Fig. 2 to Supplementary Fig. 5).

### Absolute and relative inequality scales

The information of both scales, absolute and relative, provides complementary information about public health actions. Only a moderate correlation was observed between different scales (SII and RII) in the same SES variable (rho = 0.34 for WI and rho = 0.15 for MHEL) (Supplementary Fig. 6). While absolute scale provides a measure of the public health impact of the SES inequality, the relative scale provides a measure of the strength of the association between the SES variable and the disease outcome. The only countries with a negative correlation between SII and RII for WI are Madagascar (rho = − 0.31), Angola (− 0.09), Tanzania (− 0.06), and Burundi (− 0.02). Burkina Faso, Burundi, Madagascar, Tanzania, and Kenya showed a negative correlation between SII and RII for MHEL (Supplementary Table 2).

### Spatial heterogeneity and clustering

**Figure 1:**
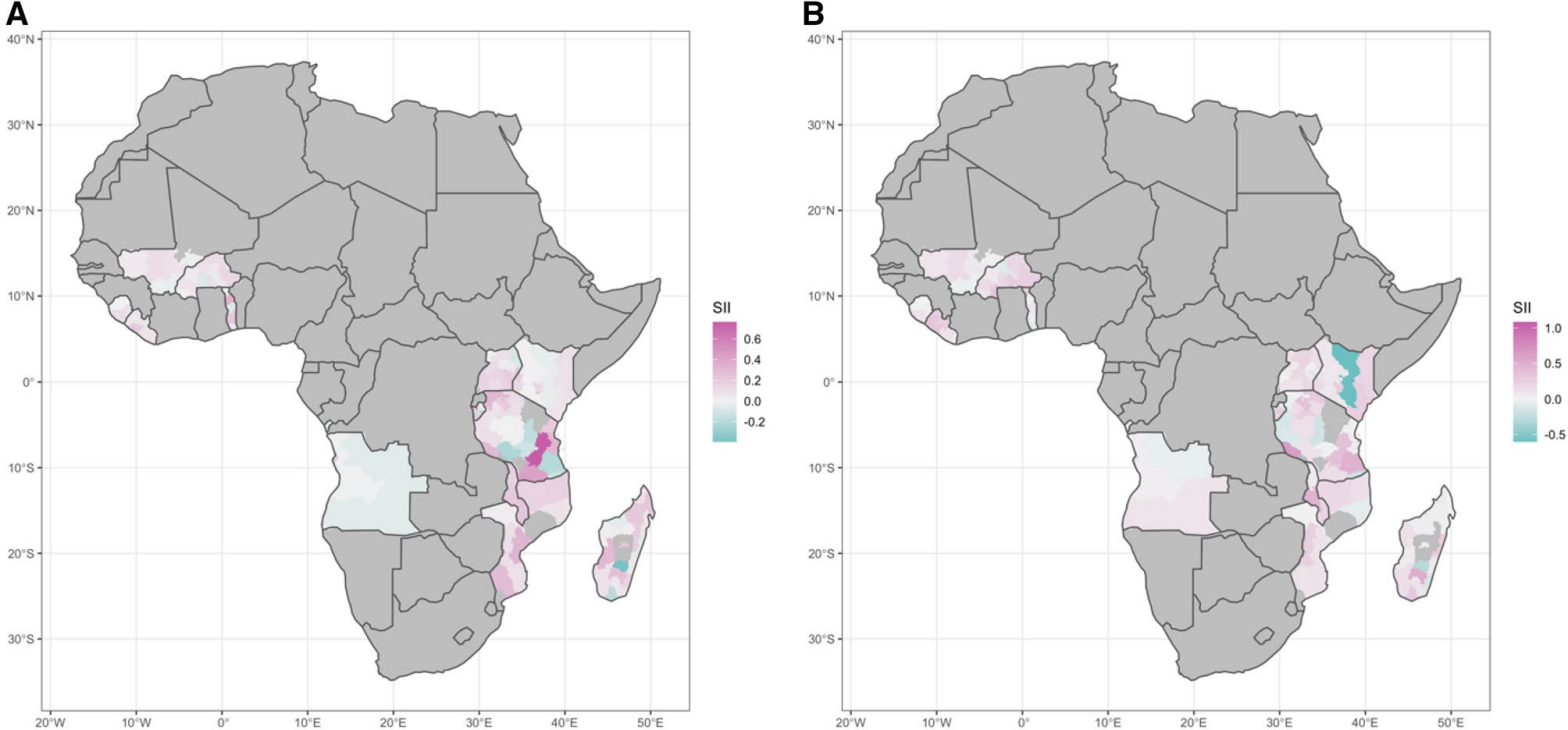
Distribution of the Slope Index of Inequality (SII) in malaria prevalence at the administrative level in Sub-Saharan African (SSA) countries. Relative to (**A**) wealth index (WI) and (**B**) mothers’ highest educational level (MHEL). The maps were generated using R software v.4.0.1 (R: A language and environment for statistical computing, R Core Team, R Foundation for Statistical Computing, Vienna, Australia (2021) http://www.R-project.org/) with ggplot2 3.3.2 (H. Wickham. ggplot2: Elegant Graphics for Data Analysis. Springer-Verlag New York, 2016.) and country boundaries from Natural Earth (https://www.naturalearthdata.com/) using the package spData 0.3.1 (https://nowosad.github.io/spData).

**Figure 2:**
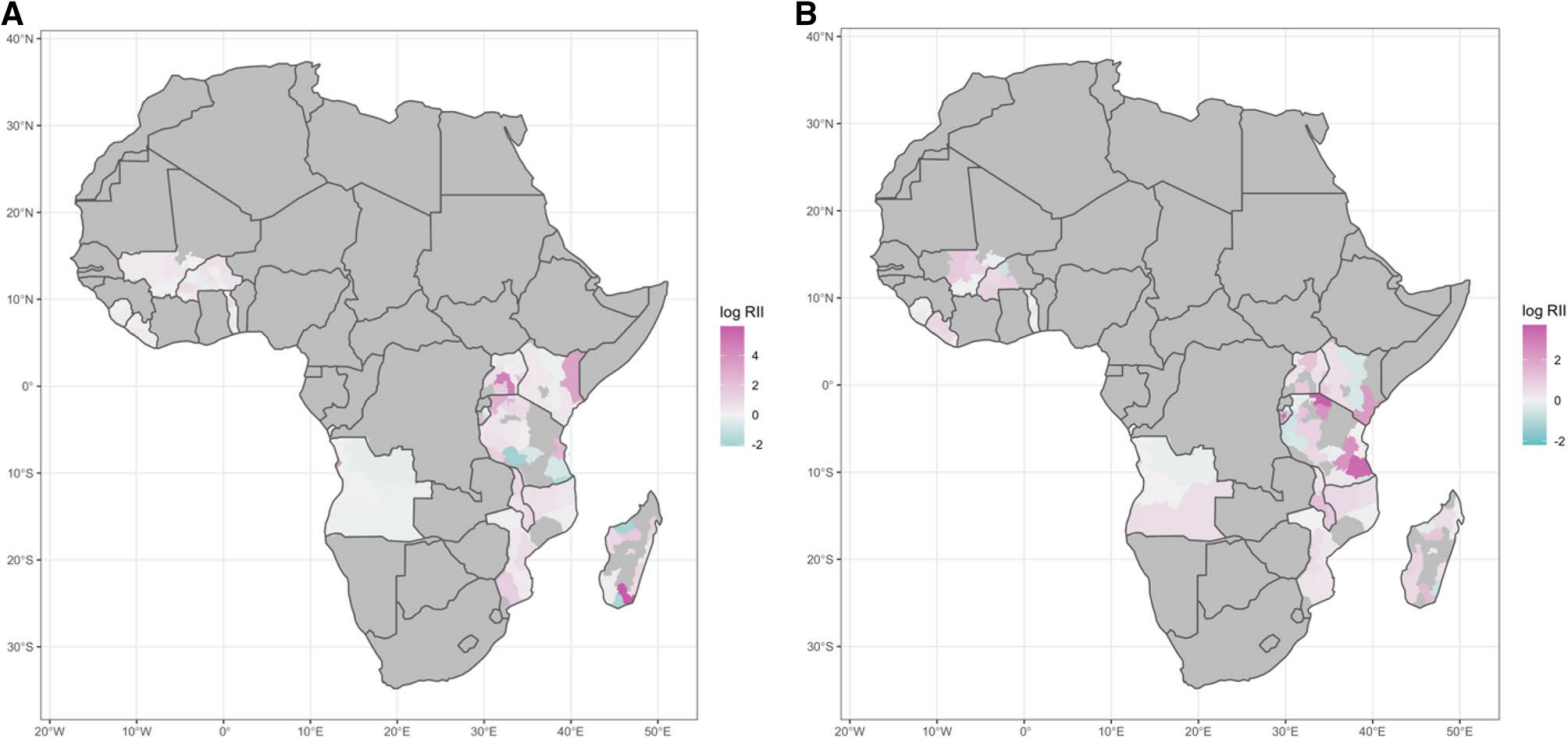
Distribution of the Relative Index of Inequality (RII) in malaria prevalence at the administrative level in Sub-Saharan African (SSA) countries. Relative to (**A**) wealth index (WI) and (**B**) mothers’ highest educational level (MHEL). RII in logarithmic scale. The maps were generated using R software v.4.0.1 (R: A language and environment for statistical computing, R Core Team, R Foundation for Statistical Computing, Vienna, Australia (2021) http://www.R-project.org/) with ggplot2 3.3.2 (H. Wickham. ggplot2: Elegant Graphics for Data Analysis. Springer-Verlag New York, 2016.) and country boundaries from Natural Earth (https://www.naturalearthdata.com/) using the package spData 0.3.1 (https://nowosad.github.io/spData).

**Figure 3:**
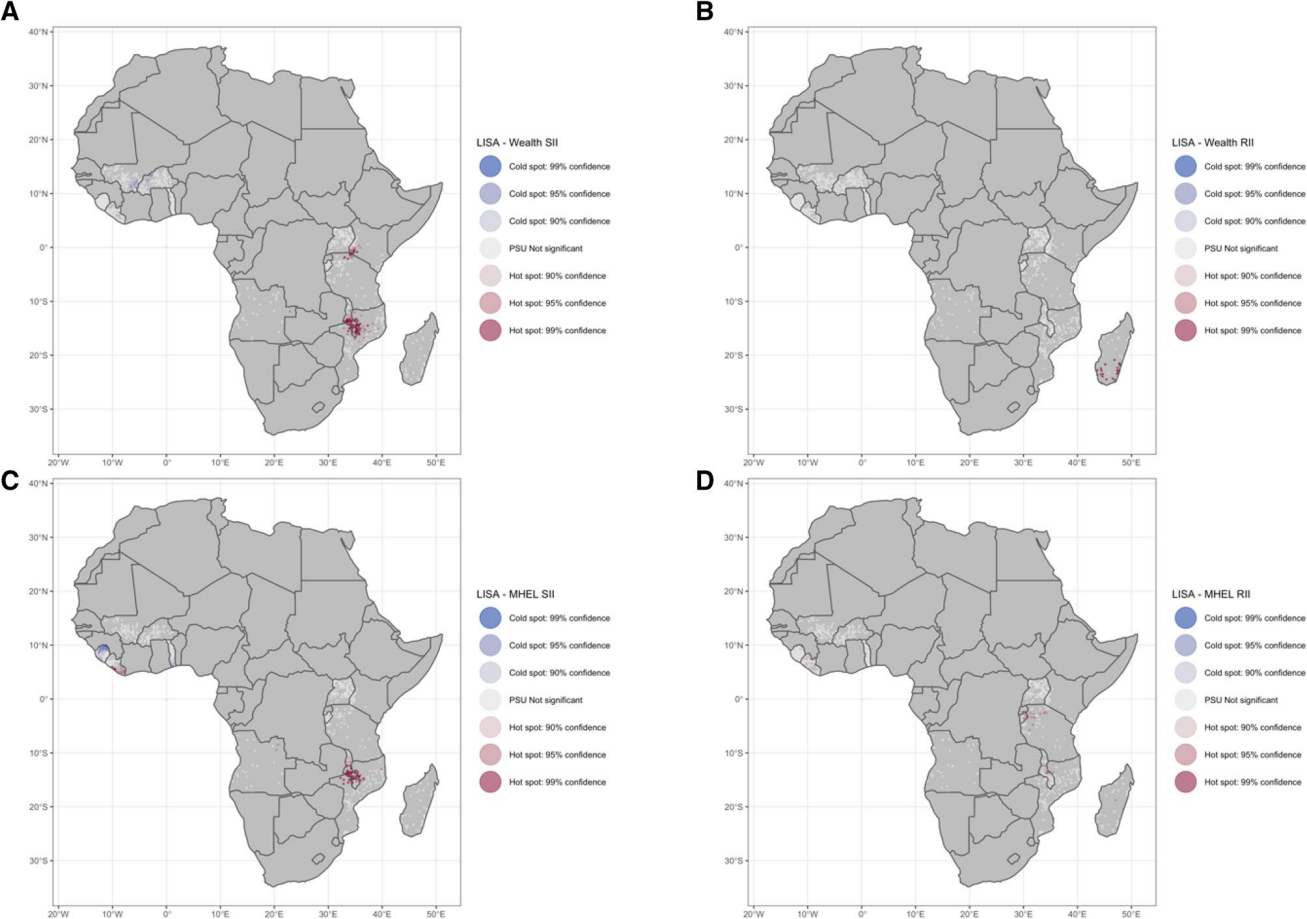
Local spatial autocorrelation of malaria inequality as Local Getis-Ord Gi* at the Primary Sampling Unit (PSU) level. Malaria inequality relative to wealth index (WI) in the (**A**) absolute and (**B**) relative scales, and relative to mothers’ highest educational level (MHEL) in the (**C**) absolute and (**D**) relative scales. The maps were generated using R software v.4.0.1 (R: A language and environment for statistical computing, R Core Team, R Foundation for Statistical Computing, Vienna, Australia (2021) http://www.R-project.org/) with ggplot2 3.3.2 (H. Wickham. ggplot2: Elegant Graphics for Data Analysis. Springer-Verlag New York, 2016.) and country boundaries from Natural Earth (https://www.naturalearthdata.com/) using the package spData 0.3.1 (https://nowosad.github.io/spData).

At the subnational level, contrasting patterns were observed between countries in SSA (Figs. 1 and 2). Countries located in Eastern Africa showed a higher median SII and RII in malaria prevalence relative to WI in comparison to countries in other locations across SSA when analyzing at administrative level (Figs. 1 and 2) and PSU level (Supplementary Fig. 7 and Supplementary Fig. 8). The clusters of high (hotspot) or low (coldspot) values of SES-inequality differ according to the SES variable and scale used (Fig. 3). Pockets of high SII in malaria prevalence in relation to WI and MHEL were observed in the East part of Africa, mainly close to Malawi and Uganda. On the other hand, pockets of low RII in malaria prevalence in relation to WI and MHEL were observed close to Burkina Faso (Fig. 3). Consistent patterns were observed when using CI as the inequality metric (Supplementary Fig. 9 to Supplementary Fig. 13).

## Discussion

This study compared two dimensions of the socioeconomic status—the wealth index and maternal education—for measuring the inequalities in malaria prevalence across Sub-Saharan African (SSA) countries using an absolute and relative scale of inequality. Our multi-country assessment provides estimations of strong socioeconomic inequalities in malaria risks between and within SSA countries. Such within- and between-countries inequalities varied greatly according to the socioeconomic metric and the scale used. Finally, this study was able to map this wide range of malaria inequality metrics at a very local scale and highlighted the spatial clustering patterns of pockets of high and low malaria inequality values.

Mapping geographical inequalities at a fine spatial scale highlights important information to guide programmatic actions that would help reduce malaria inequalities, such as targeted provision of sanitation or control of infectious diseases^48–50^. Despite sparse studies that highlighted important socioeconomic inequalities regarding malaria prevalence in different contexts^22,23,25^, a comprehensive assessment of the malaria inequality landscape in SSA countries were not available. Understanding such malaria inequalities and their localization at a very fine scale is of particular interest in SSA where multiple contemporary challenges might synergistically impact in vulnerable populations and worsen their health status.

This paper constitutes the first assessment of malaria SES inequalities across many SSA countries while assessing the spatial distribution of such malaria inequalities. We emphasize that the malaria burden is not shared equally across SES strata in SSA (both within and between countries) and that such inequalities are not randomly distributed across space. We believe this work could be extremely beneficial for existing programs to incorporate an inequality focus into malaria prevention efforts and identify areas (at a fine geographical scale) where such inequalities are concentrated for informing targeted efforts. The results presented here showed the important and complementary information of the multiple dimensions of socioeconomic status. The fine-scale mapping showed in this study stressed out the contrasting spatial patterns of malaria inequalities in relation to wealth or maternal education. Previous studies showed that both maternal education and wealth strongly influence knowledge about and efforts to prevent and treat malaria in Madagascar^51^. Furthermore, a recent systematic review suggest that public policy measures that can improve economic and educational opportunities for the poor, will help in reducing the burden of malaria in SSA^14^. In addition, a multi-country study highlighted that maternal education and wealth are important components of a simplified SES index^52^. Taken together, the results of this study emphasize the multiple socioeconomic mechanisms that may impact in the uneven malaria distribution also at a very local level.

This study has a number of limitations. First, socioeconomic metrics such as the components of the wealth index and maternal education may be subject to measurement error due to recall bias, inaccurate reporting during interviews, and social desirability bias. Second, despite the fine spatial scale of this study, these results might not fully represent intra-urban malaria inequalities. Third, our analysis does not capture the impacts of conflicts or climate change-related meteorological events and disasters, and data for locations affected by these factors might not reflect current or seasonal conditions. Fourth, the spatial jittering injected to PSU’s GPS data (to preserve confidential data) may affect the spatial clustering analysis.

In conclusion, socioeconomic inequality of malaria in relation to wealth index and maternal education were strongly heterogeneous with pockets of high and low malaria inequality across SSA. The use of multiple metrics of socioeconomic status and scales of inequality metrics allows for a better characterization of the multiple mechanisms in place that originate such strong malaria heterogeneities observed in this study. Finally, historical malaria inequalities reflected in this study collide with current challenges that might synergistically and negatively impacts the health of vulnerable populations in SSA countries.

## Supplementary Information

Supplementary Information.

## Data Availability

The datasets used in this study are available upon authorization from the DHS program and can be accessed on the website (https://dhsprogram.com/data/available-datasets.cfm).

## Code availability

Codes to reproduce the inequalities indicators, the figures, and tables can be accessed at https://bookdown.org/gabc91/mal_ineq/.
