# Supplementary material for: Mapping socioeconomic inequalities in malaria in Sub-Sahara African countries
Source: Sci Rep. 2021 Jul 23;11:15121. doi: 10.1038/s41598-021-94601-x (PMC8302762; doi:10.1038/s41598-021-94601-x)
Supplement: Supplementary file 1 — Supplementary Information. [file 41598_2021_94601_MOESM1_ESM.docx]

**Mapping socioeconomic inequalities in malaria in Sub-Sahara African countries**

Gabriel Carrasco-Escobar^1,2,*^, Kimberly Fornace^3^, Tarik Benmarhnia^2,4^

**Author Affiliated Institutions:**

^1^ Health Innovation Lab, Institute of Tropical Medicine “Alexander von Humboldt”, Universidad Peruana Cayetano Heredia, Lima, Peru

^2^ Scripps Institution of Oceanography, University of California, San Diego, CA, USA

^3^ Faculty of Infectious and Tropical Diseases, London School of Hygiene and Tropical Medicine, London, United Kingdom

^4^ Department of Family Medicine and Public Health, University of California, San Diego, CA, USA

*** Corresponding author:** Gabriel Carrasco Escobar, MSc: [gabriel.carrasco@upch.pe](mailto:gabriel.carrasco@upch.pe)

**SUPPLEMENTARY INFORAMTION**

1. **Supplementary Methods**
   1. **Socio-economic inequality analysis**

We additionally assessed the socio-economic inequalities at the Primary Sampling Unit (PSU) and administrative level by computing the concentration index (CI) relative to wealth index (WI) and mothers’ highest educational level (MHEL). The CI is a measure of inequalities^1^ that represents the cumulative proportion of malaria relative to individuals ranked by SES levels (such as WI and MHEL). CI ranges from -1 to +1, where negative values represent uneven concentration of malaria among low-SES levels and positive values represent high malaria concentration among high-SES levels.

- 1. **References**

1. Wagstaff, A., Paci, P. & van Doorslaer, E. On the measurement of inequalities in health. *Soc Sci Med* **33**, 545–557 (1991).

1. **Supplementary Tables**

**Supplementary Table 1.** Correlation (Spearman rho) between socioeconomic metrics (wealth index – WI and mothers’ highest educational level – MHEL) of the same inequality scale (Slope Index of Inequality **–** SII or Relative Index of Inequality **–** RII).

| **country** | **SII**  **(WI vs. MHEL)** | **RII**  **(WI vs. MHEL)** |
| --- | --- | --- |
| **AO** | 0.135994 | 0.326387 |
| **BF** | 0.0985 | -0.06366 |
| **BU** | 0.180475 | -0.06448 |
| **KE** | -0.01379 | -0.03258 |
| **LB** | 0.240327 | 0.185114 |
| **MD** | 0.036321 | 0.063119 |
| **ML** | 0.053005 | 0.081587 |
| **MW** | 0.194339 | 0.196108 |
| **MZ** | 0.077924 | 0.163046 |
| **SL** | 0.069832 | 0.193409 |
| **TG** | 0.061585 | 0.120461 |
| **TZ** | 0.188967 | 0.305895 |
| **UG** | 0.079249 | 0.234196 |

**Supplementary Table 2.** Correlation (Spearman rho) between inequality scales (Slope Index of Inequality **–** SII and Relative Index of Inequality – RII) of the same socioeconomic metric (wealth index – WI or mothers’ highest educational level – MHEL).

| **country** | **WI**  **(SII vs. RII)** | **MHEL**  **(SII vs. RII)** |
| --- | --- | --- |
| **AO** | -0.0862905 | 0.1780595 |
| **BF** | 0.14886699 | -0.3951633 |
| **BU** | -0.0255866 | -0.1015101 |
| **KE** | 0.67658442 | -0.1504254 |
| **LB** | 0.61835933 | 0.53521058 |
| **MD** | -0.3190823 | -0.0033401 |
| **ML** | 0.45246805 | 0.07631938 |
| **MW** | 0.30076445 | 0.15252611 |
| **MZ** | 0.43270236 | 0.30120118 |
| **SL** | 0.85740086 | 0.46854434 |
| **TG** | 0.3007293 | 0.30342344 |
| **TZ** | -0.0671741 | -0.0180846 |
| **UG** | 0.39863396 | 0.17344005 |

1. **Supplementary Figures**

**Supplementary Figure 1.** Distribution of Primary Sampling Unit (PSU) A) sample size, B) malaria prevalence, and C) their relation.


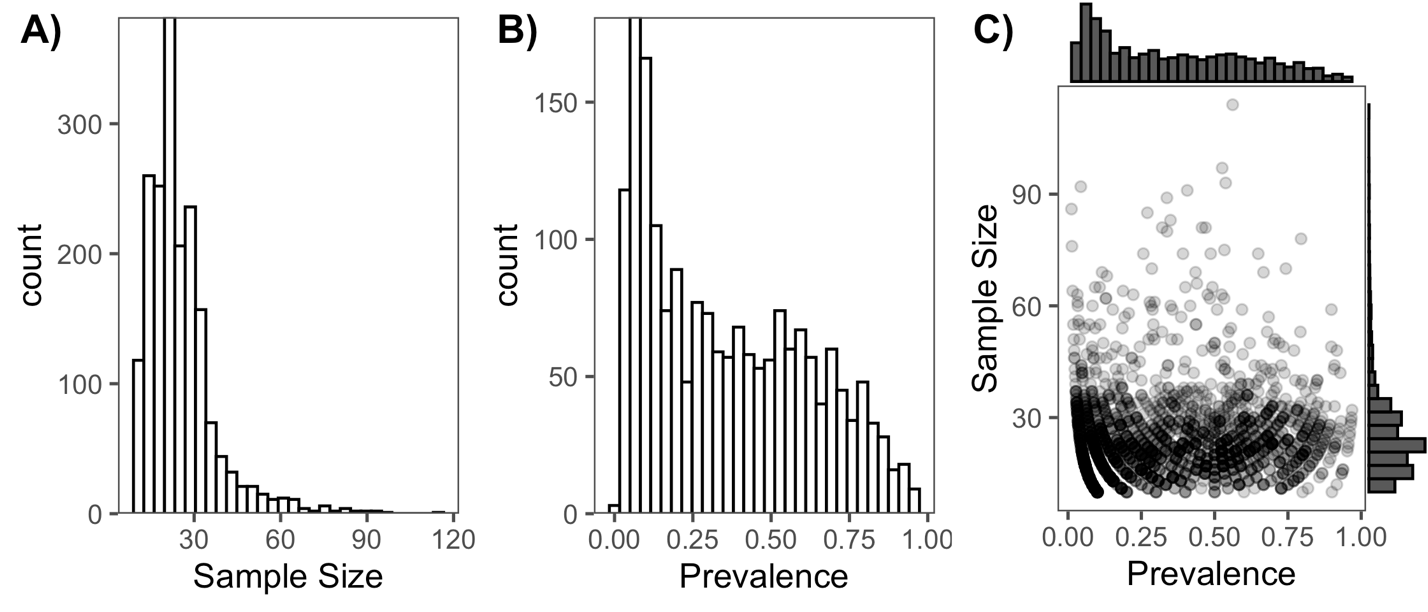


**Supplementary Figure 2. Sensitivity analysis of the distribution of the Primary Sampling Unit (PSU)-level Slope Index of Inequality (SII) in malaria prevalence relative to wealth index (WI).** Left column: Distribution of SII across thirteen Sub-Saharan African (SSA) countries including only PSUs with a sample size higher than A) 10, B) 20, C) and 30. Each point represents a PSU. Points were horizontally scattered for visualization purposes. Right column: Distribution of SII relative to the malaria prevalence in each PSU including only PSUs with a sample size higher than A) 10, B) 20, C) and 30. Point radius relative to the sample size at the PSU. Re-analysis was conducted on sub-samples with PSU sample size larger than 20 and 30 participants.


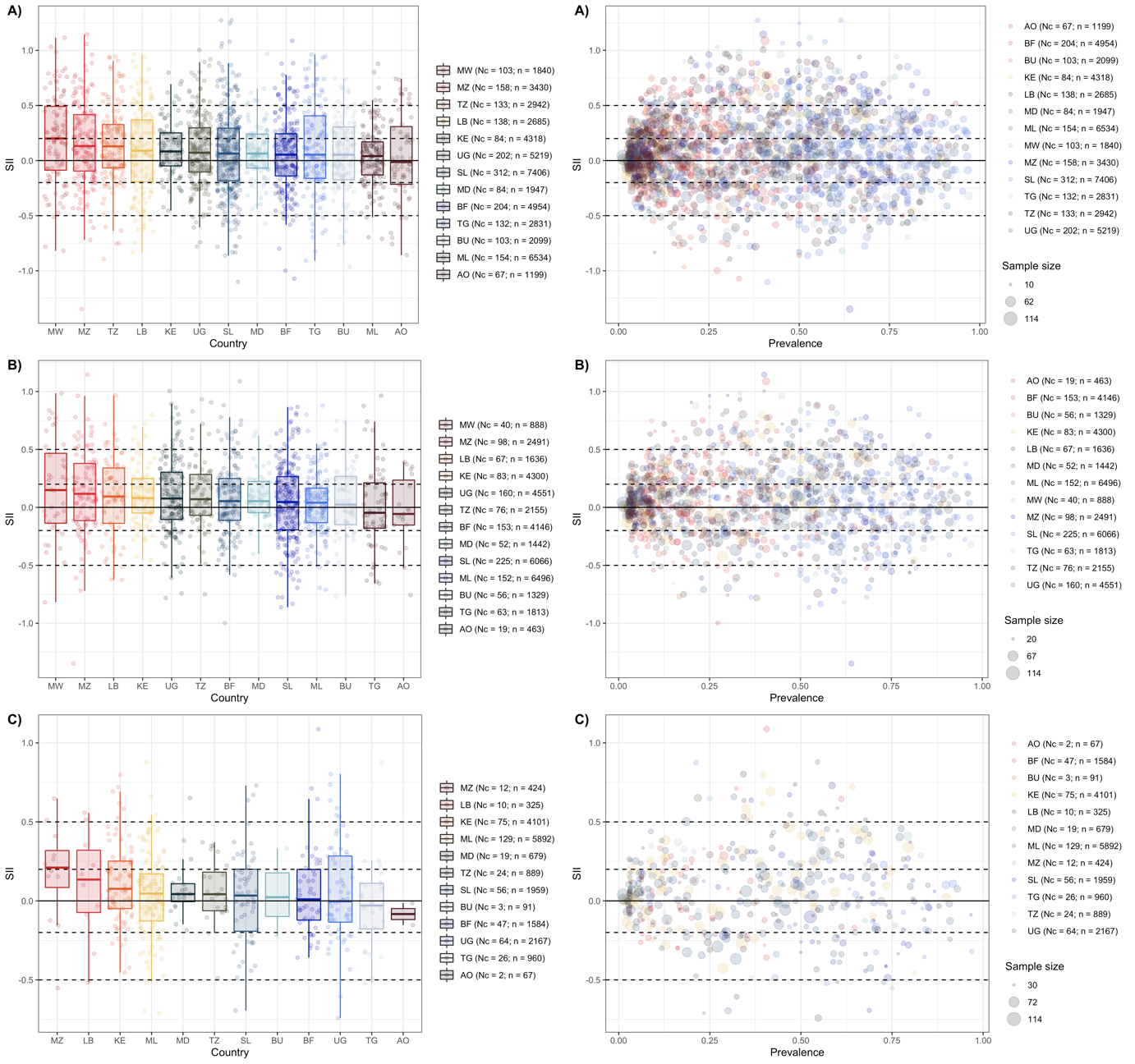


**Supplementary Figure 3. Sensitivity analysis of the distribution of the Primary Sampling Unit (PSU)-level Relative Index of Inequality (RII) in malaria prevalence relative to wealth index (WI).** Left column: Distribution of RII across thirteen Sub-Saharan African (SSA) countries including only PSUs with a sample size higher than A) 10, B) 20, C) and 30. Each point represents a PSU. Points were horizontally scattered for visualization purposes. Right column: Distribution of RII relative to the malaria prevalence in each PSU including only PSUs with a sample size higher than A) 10, B) 20, C) and 30. Point radius relative to the sample size at the PSU. Re-analysis was conducted on sub-samples with PSU sample size larger than 20 and 30 participants. Y-axis in log-scale.


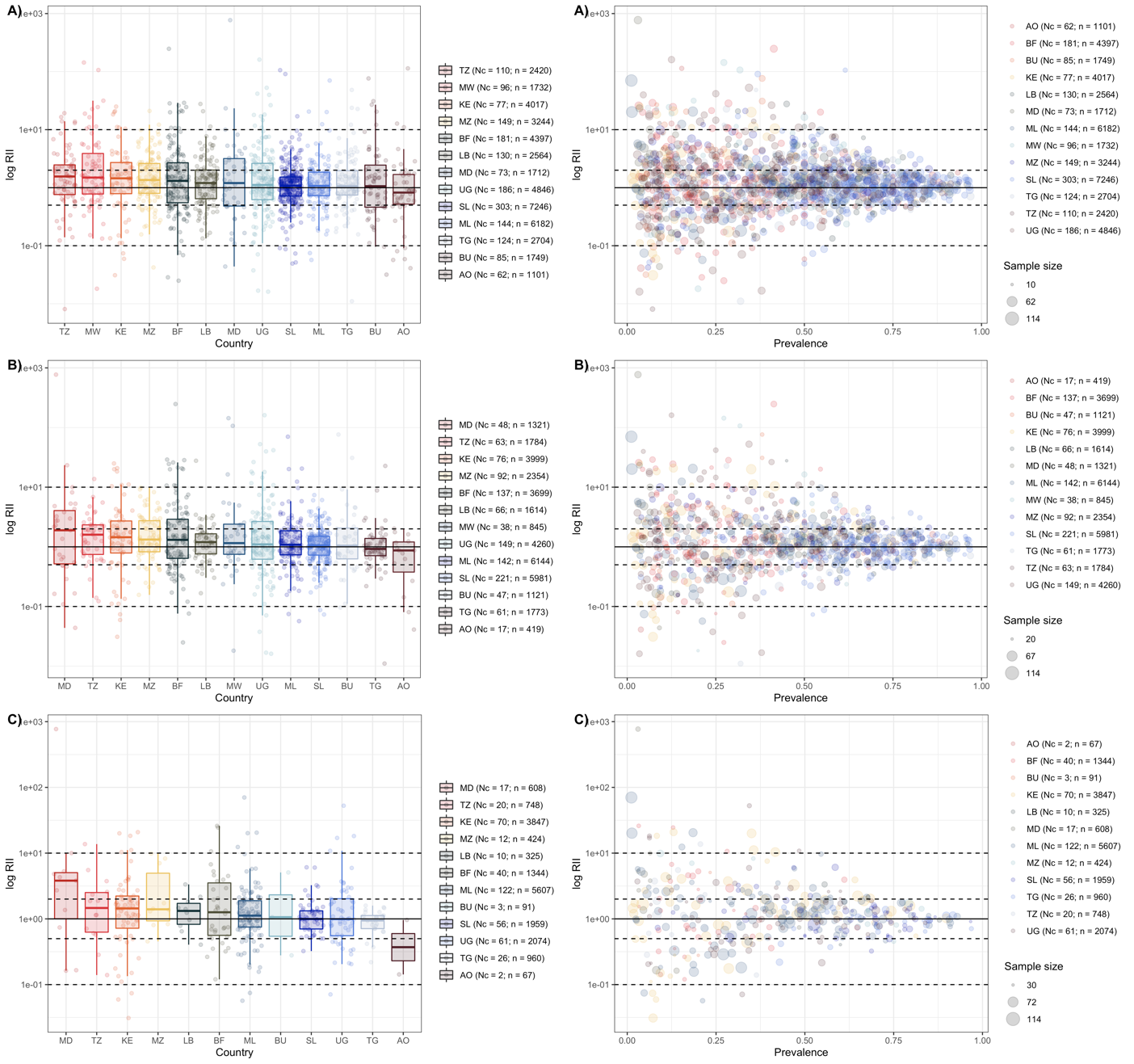


**Supplementary Figure 4. Sensitivity analysis of the distribution of the Primary Sampling Unit (PSU)-level Slope Index of Inequality (SII) in malaria prevalence relative to mothers’ highest educational level (MHEL).** Left column: Distribution of SII across thirteen Sub-Saharan African (SSA) countries including only PSUs with a sample size higher than A) 10, B) 20, C) and 30. Each point represents a PSU. Points were horizontally scattered for visualization purposes. Right column: Distribution of SII relative to the malaria prevalence in each PSU including only PSUs with a sample size higher than A) 10, B) 20, C) and 30. Point radius relative to the sample size at the PSU. Re-analysis was conducted on sub-samples with PSU sample size larger than 20 and 30 participants.


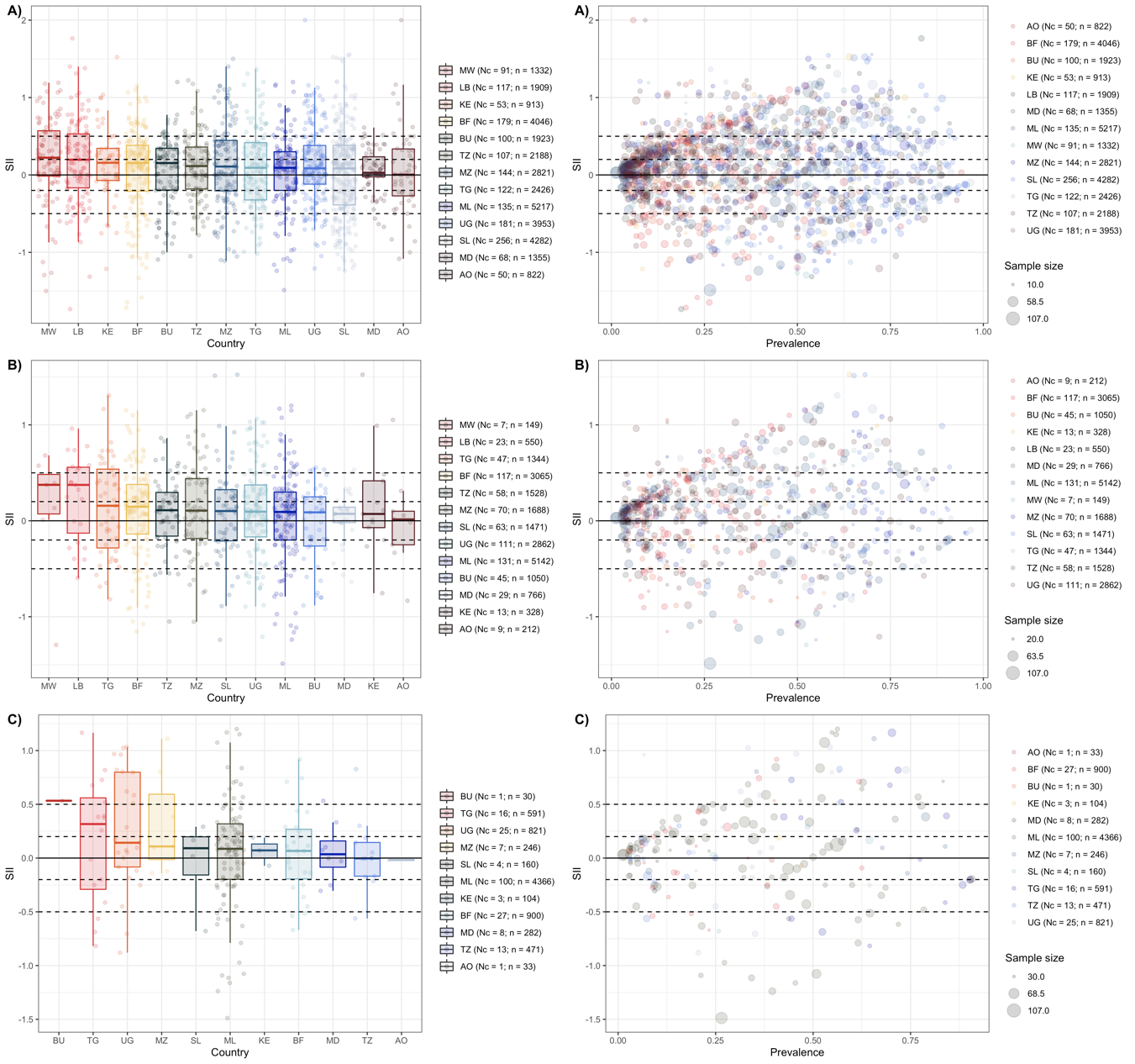


**Supplementary Figure 5. Sensitivity analysis of the distribution of the Primary Sampling Unit (PSU)-level Relative Index of Inequality (RII) in malaria prevalence relative to mothers’ highest educational level (MHEL).** Left column: Distribution of RII across thirteen Sub-Saharan African (SSA) countries including only PSUs with a sample size higher than A) 10, B) 20, C) and 30. Each point represents a PSU. Points were horizontally scattered for visualization purposes. Right column: Distribution of RII relative to the malaria prevalence in each PSU including only PSUs with a sample size higher than A) 10, B) 20, C) and 30. Point radius relative to the sample size at the PSU. Re-analysis was conducted on sub-samples with PSU sample size larger than 20 and 30 participants. Y-axis in log-scale.


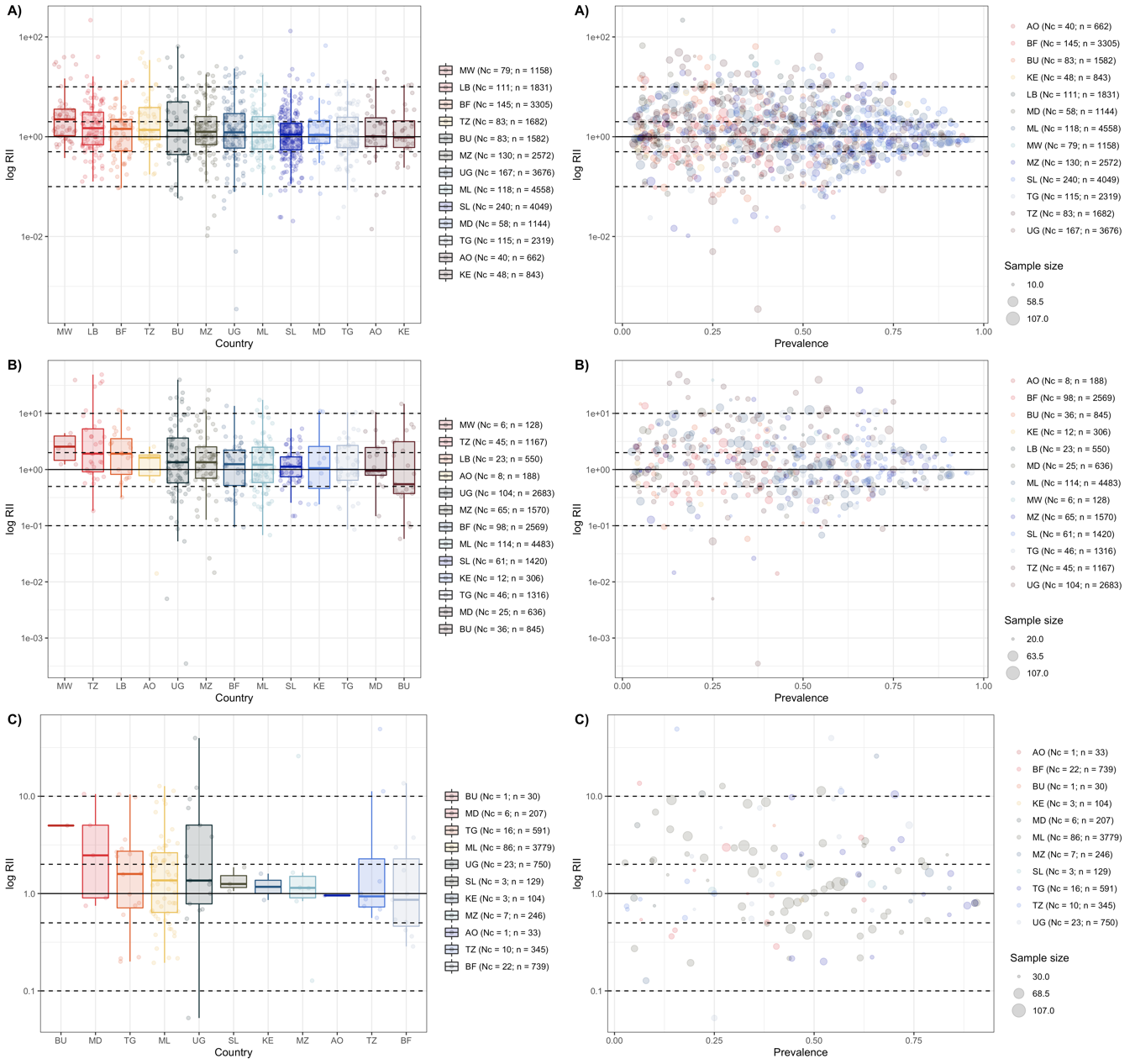


**Supplementary Figure 6. Distribution and correlation between the Slope Index of Inequality (SII) and the log Relative Index of Inequality (RII).** In relation to A) Wealth Index (WI), and B) Mothers; Highest Educational Level (MHEL). Each point represents a Primary Sampling Unit (PSU).


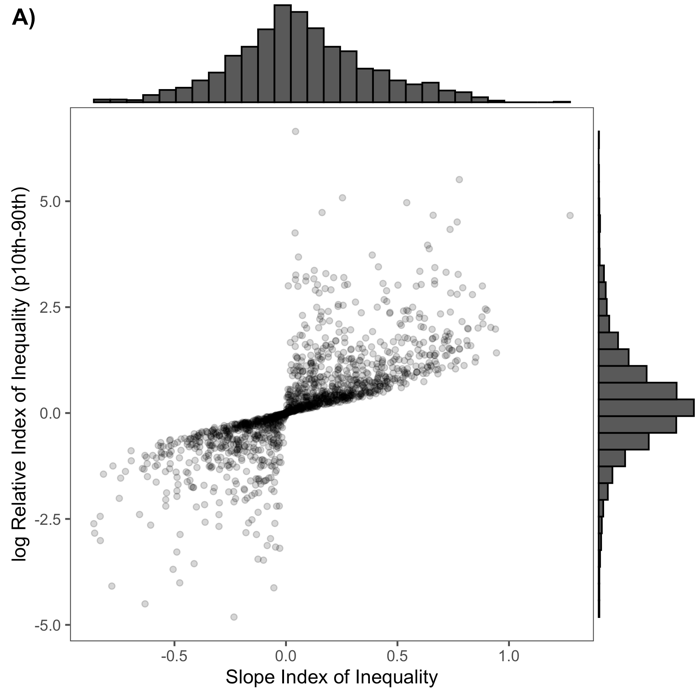

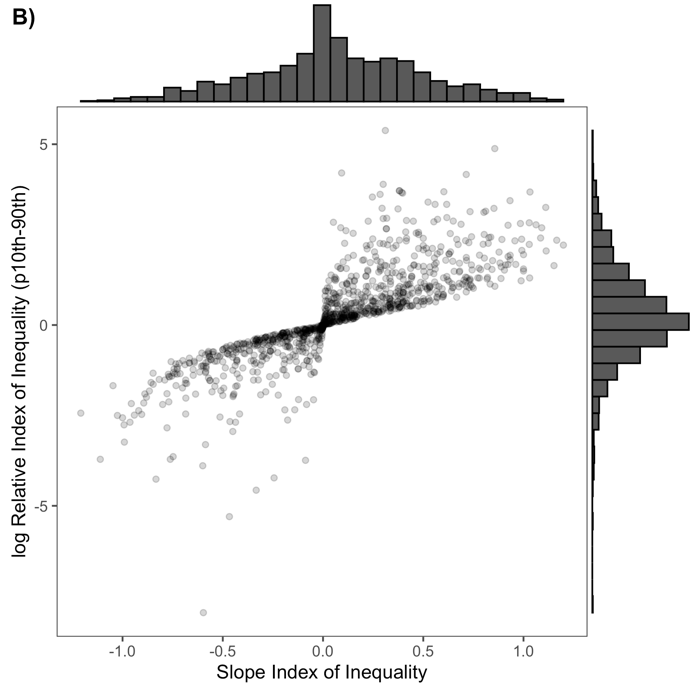


**Supplementary Figure 7. Distribution of the Slope Index of Inequality (SII) in malaria prevalence at the Primary Sampling Unit (PSU) level in Sub-Saharan African (SSA) Countries.** Relative to A) wealth index (WI) and B) mothers’ highest educational level (MHEL). The maps were generated using R software v.4.0.1 (R: A language and environment for statistical computing, R Core Team, R Foundation for Statistical Computing, Vienna, Australia (2021) <http://www.R-project.org/>) with ggplot2 3.3.2 (H. Wickham. ggplot2: Elegant Graphics for Data Analysis. Springer-Verlag New York, 2016.) and country boundaries from Natural Earth (<https://www.naturalearthdata.com/>) using the package spData 0.3.1 (https://nowosad.github.io/spData).

**
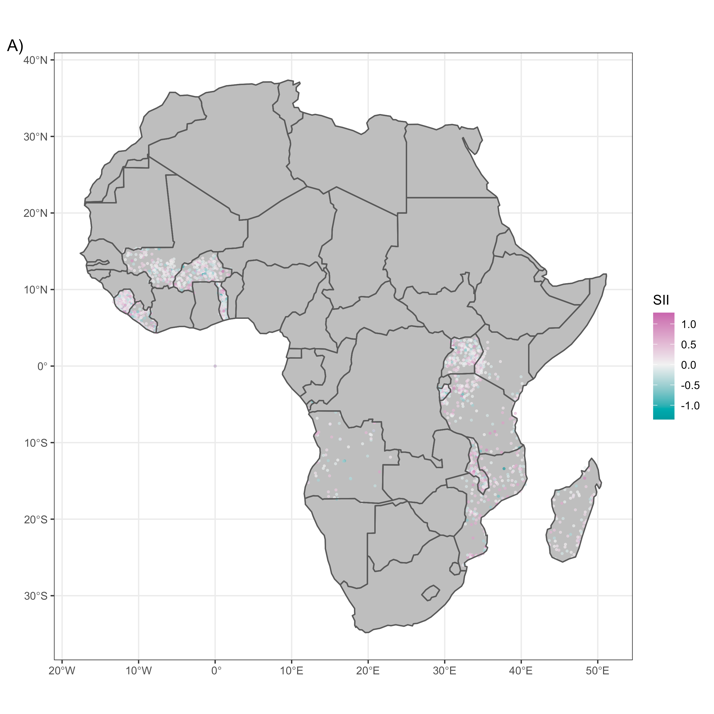

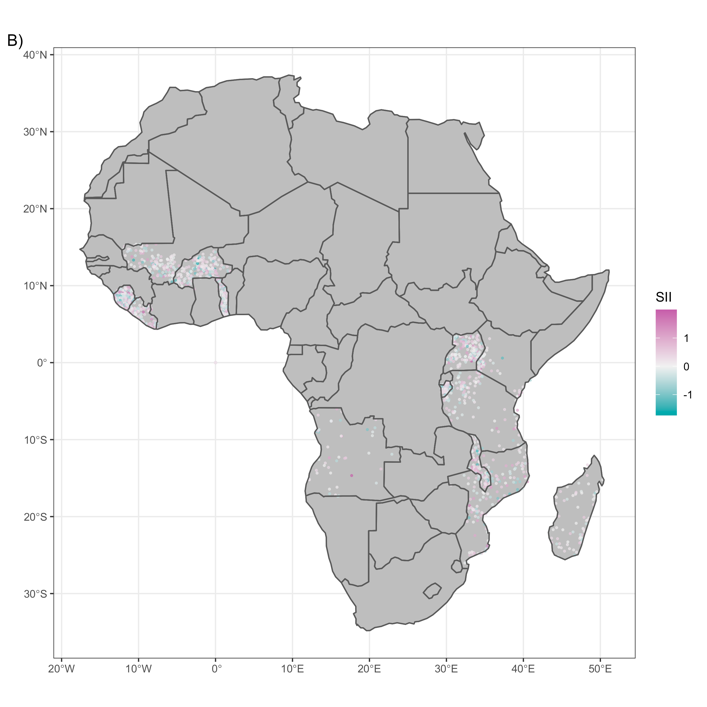
**

**Supplementary Figure 8. Distribution of the Relative Index of Inequality (RII) in malaria prevalence at the Primary Sampling Unit (PSU) level in Sub-Saharan African (SSA) Countries.** Relative to A) wealth index (WI) and B) mothers’ highest educational level (MHEL). The maps were generated using R software v.4.0.1 (R: A language and environment for statistical computing, R Core Team, R Foundation for Statistical Computing, Vienna, Australia (2021) <http://www.R-project.org/>) with ggplot2 3.3.2 (H. Wickham. ggplot2: Elegant Graphics for Data Analysis. Springer-Verlag New York, 2016.) and country boundaries from Natural Earth (<https://www.naturalearthdata.com/>) using the package spData 0.3.1 (https://nowosad.github.io/spData).

**
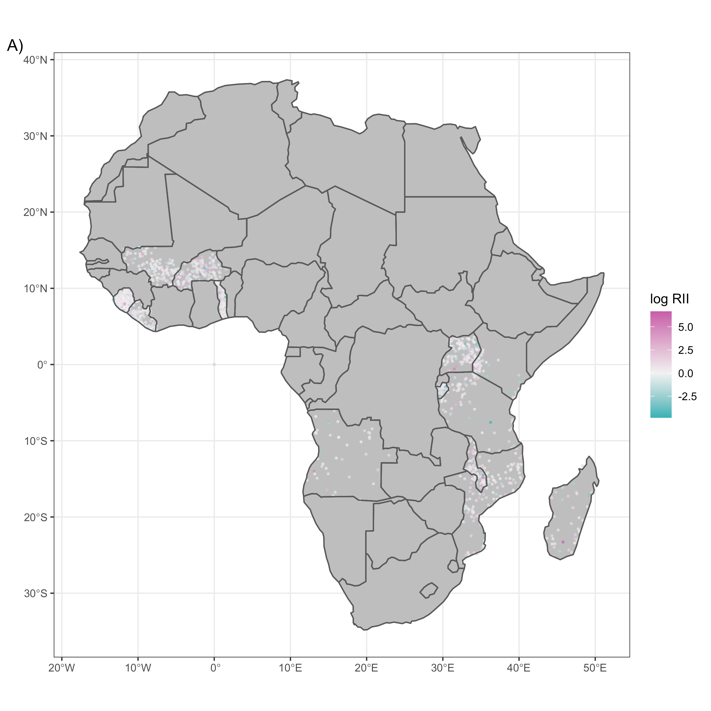

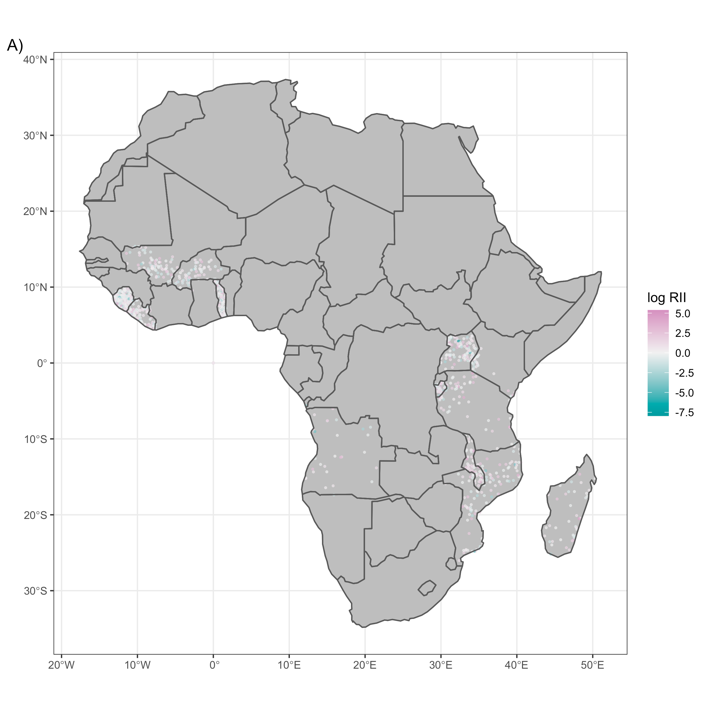
**

**Supplementary Figure 9. Distribution of the Concentration Index (CI) in malaria prevalence relative to wealth index (WI).** A) Spatial distribution at the Primary Sampling Unit (PSU) level. B) Spatial distribution at the administrative level. The maps were generated using R software v.4.0.1 (R: A language and environment for statistical computing, R Core Team, R Foundation for Statistical Computing, Vienna, Australia (2021) <http://www.R-project.org/>) with ggplot2 3.3.2 (H. Wickham. ggplot2: Elegant Graphics for Data Analysis. Springer-Verlag New York, 2016.) and country boundaries from Natural Earth (<https://www.naturalearthdata.com/>) using the package spData 0.3.1 (https://nowosad.github.io/spData).


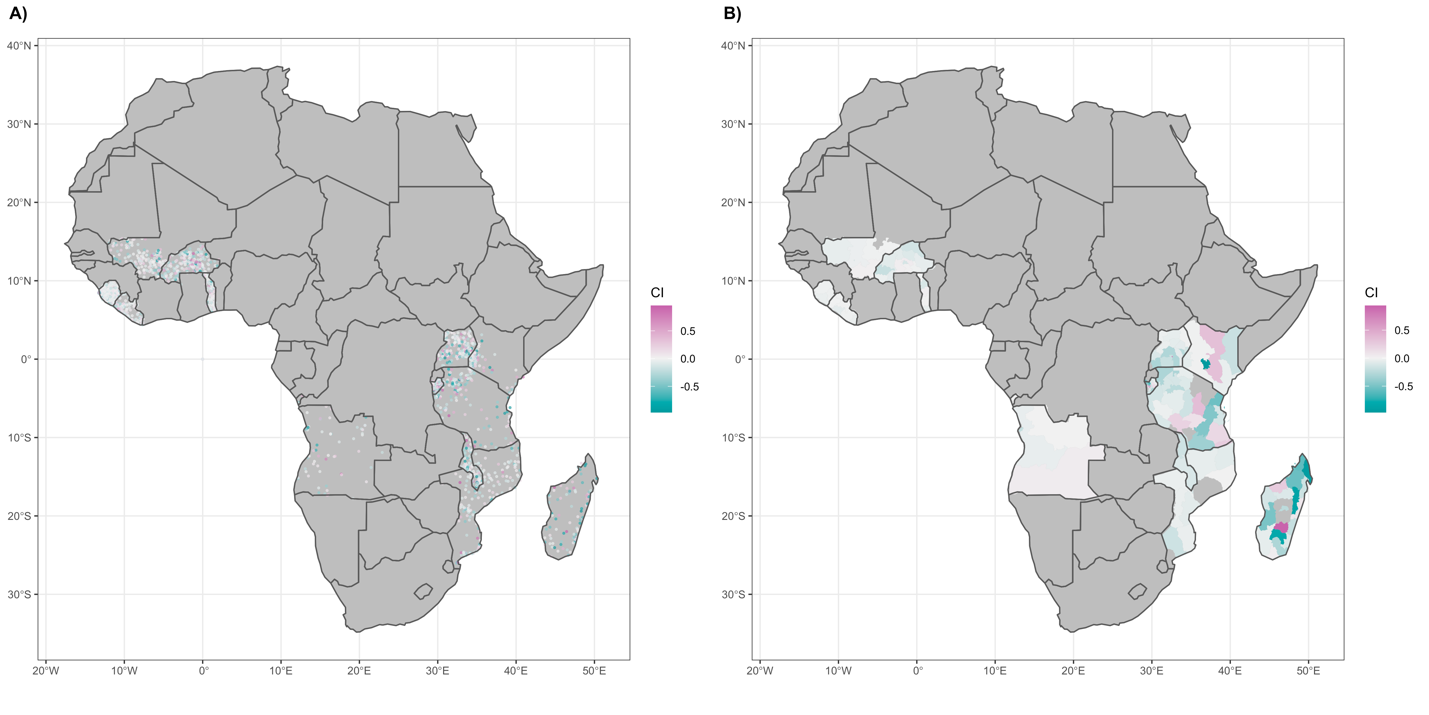


**Supplementary Figure 10. Sensitivity analysis of the distribution of the Primary Sampling Unit (PSU)-level Concentration Index (CI) in malaria prevalence relative to wealth index (WI).** Left column: Distribution of CI across thirteen Sub-Saharan African (SSA) countries including only PSUs with a sample size higher than A) 10, B) 20, C) and 30. Each point represents a PSU. Points were horizontally scattered for visualization purposes. Right column: Distribution of CI relative to the malaria prevalence in each PSU including only PSUs with a sample size higher than A) 10, B) 20, C) and 30. Point radius relative to the sample size at the PSU. Re-analysis was conducted on sub-samples with PSU sample size larger than 20 and 30 participants.


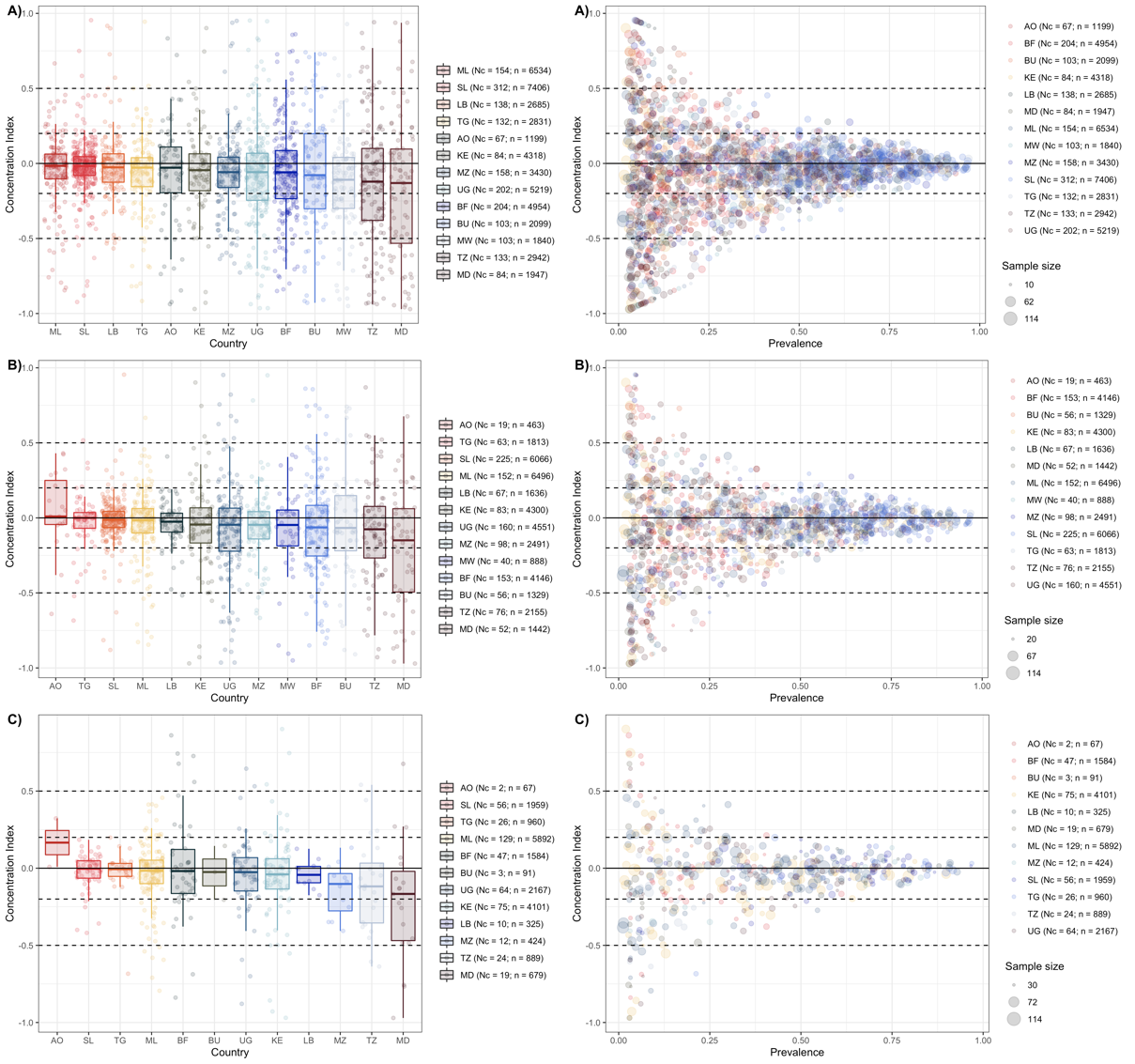


**Supplementary Figure 11. Distribution of the Concentration Index (CI) in malaria prevalence relative to mothers’ highest educational level (MHEL).** A) Spatial distribution at the Primary Sampling Unit (PSU) level. B) Spatial distribution at the administrative level. The maps were generated using R software v.4.0.1 (R: A language and environment for statistical computing, R Core Team, R Foundation for Statistical Computing, Vienna, Australia (2021) <http://www.R-project.org/>) with ggplot2 3.3.2 (H. Wickham. ggplot2: Elegant Graphics for Data Analysis. Springer-Verlag New York, 2016.) and country boundaries from Natural Earth (<https://www.naturalearthdata.com/>) using the package spData 0.3.1 (https://nowosad.github.io/spData).

**
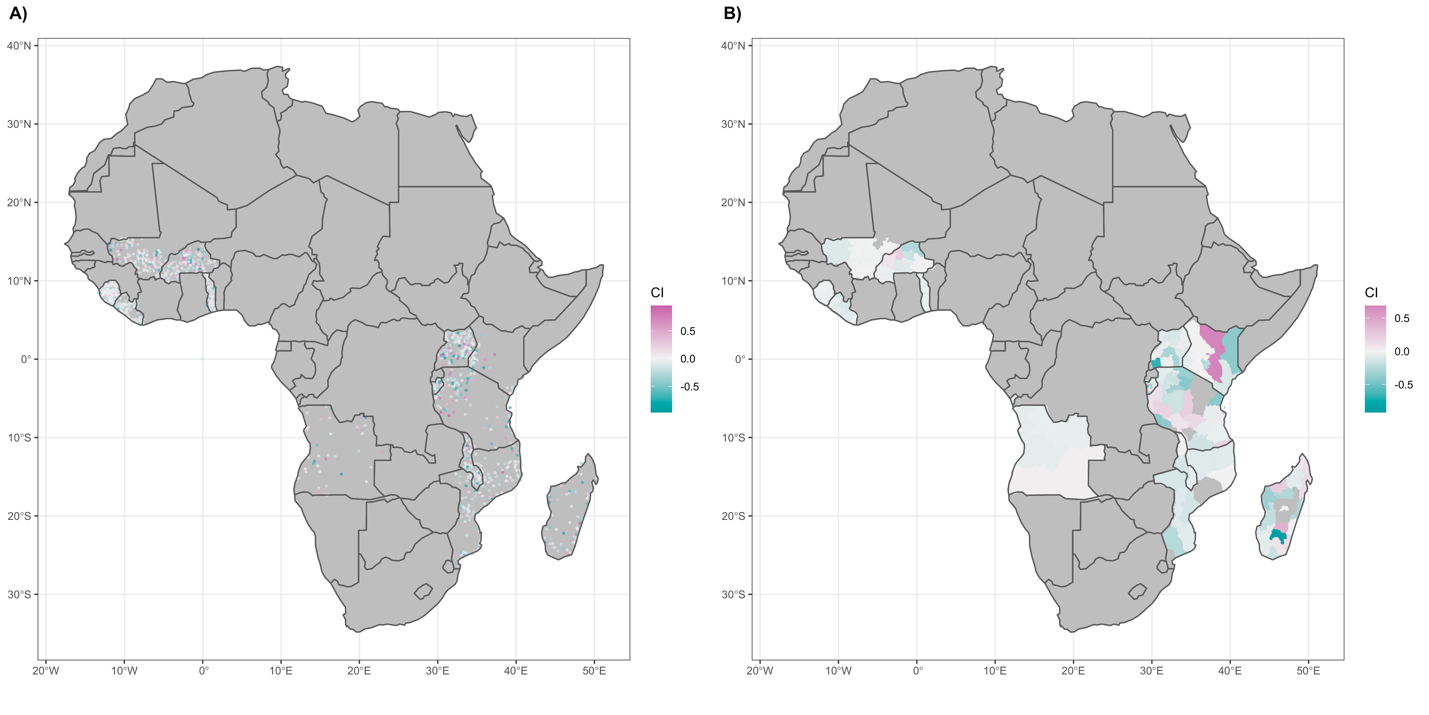
**

**Supplementary Figure 12. Sensitivity analysis of the distribution of the Primary Sampling Unit (PSU)-level Concentration Index (CI) in malaria prevalence relative to mothers’ highest educational level (MHEL).** Left column: Distribution of CI across thirteen Sub-Saharan African (SSA) countries including only PSUs with a sample size higher than A) 10, B) 20, C) and 30. Each point represents a PSU. Points were horizontally scattered for visualization purposes. Right column: Distribution of CI relative to the malaria prevalence in each PSU including only PSUs with a sample size higher than A) 10, B) 20, C) and 30. Point radius relative to the sample size at the PSU. Re-analysis was conducted on sub-samples with PSU sample size larger than 20 and 30 participants.


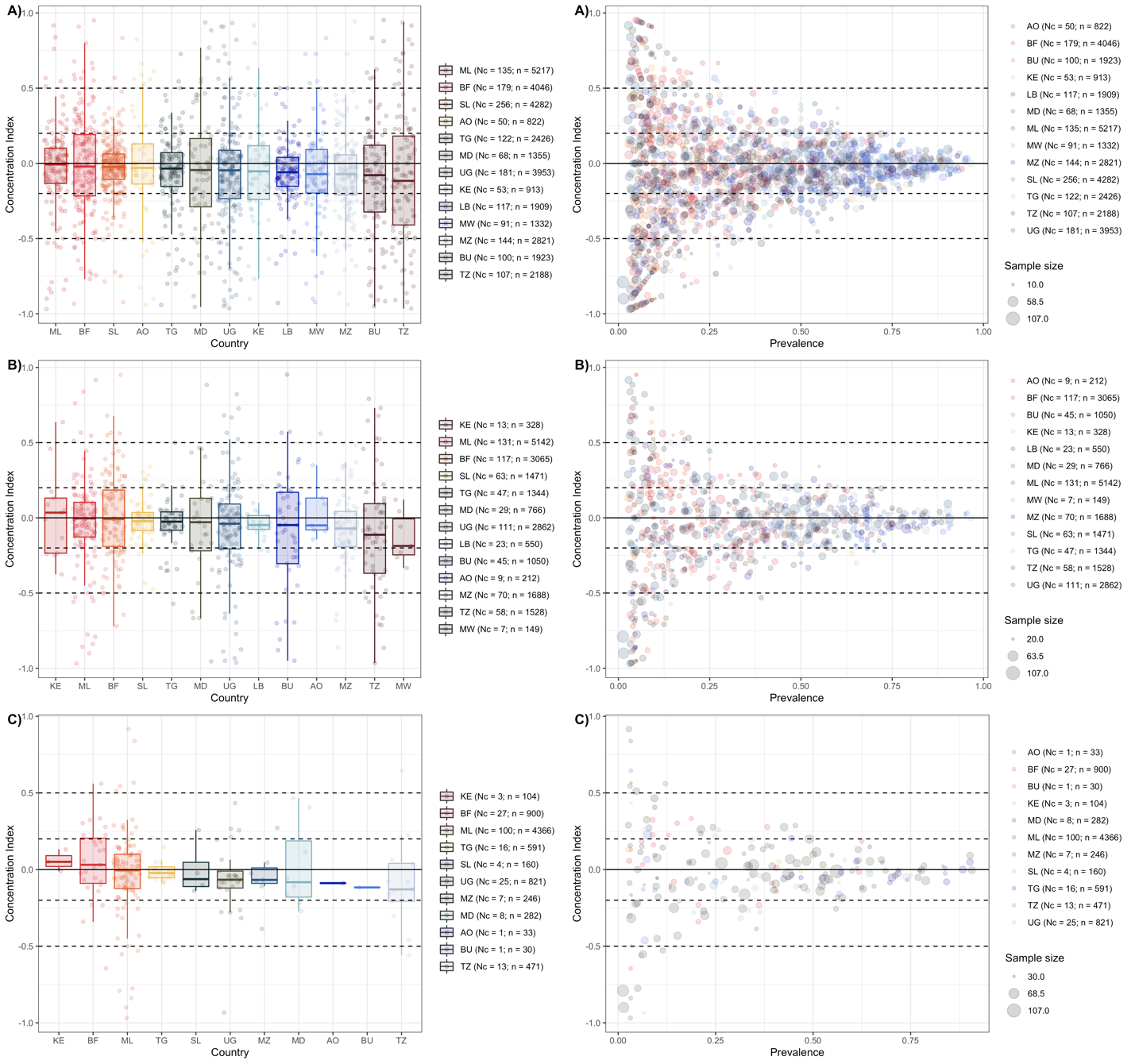


**Supplementary Figure 13. Local spatial autocorrelation of malaria inequality as Local Getis-Ord Gi*.** Malaria inequality concentration index (CI) relative to A) wealth index (WI) and B) mothers’ highest educational level (MHEL). The maps were generated using R software v.4.0.1 (R: A language and environment for statistical computing, R Core Team, R Foundation for Statistical Computing, Vienna, Australia (2021) <http://www.R-project.org/>) with ggplot2 3.3.2 (H. Wickham. ggplot2: Elegant Graphics for Data Analysis. Springer-Verlag New York, 2016.) and country boundaries from Natural Earth (<https://www.naturalearthdata.com/>) using the package spData 0.3.1 (https://nowosad.github.io/spData).

**
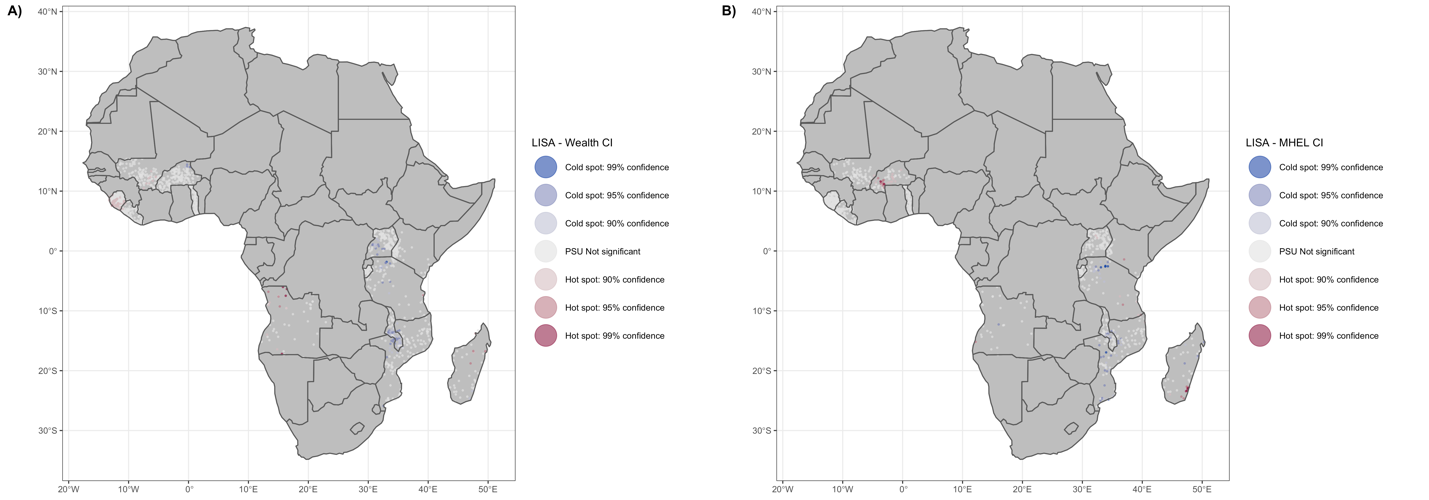
**
